# Long-term traditional Chinese medicine use and incidence of metabolic syndrome in schizophrenia: a retrospective cohort study

**DOI:** 10.3389/fphar.2026.1715482

**Published:** 2026-03-17

**Authors:** Jing-Shuang Zhang, Wei-Jun Gu, Ying-Hua Huang, Mei Guo

**Affiliations:** Xiamen Xianyue Hospital, Xianyue Hospital Affiliated with Xiamen Medical College, Fujian Psychiatric Center, Fujian Clinical Research Center for Mental Disorders, Xiamen, China

**Keywords:** antipsychotic drugs, metabolic syndrome (MetS), retrospective cohort study, schizophrenia, traditional Chinese medicine (TCM)

## Abstract

**Objective:**

This study aims to assess the impact of medium-to long-term traditional Chinese medicine (TCM) combined with antipsychotic medications on metabolic syndrome (MetS) in patients with schizophrenia and to explore the prevalence and related influencing factors of MetS in these patients.

**Methods:**

A retrospective cohort study was conducted among hospitalized patients in a psychiatric hospital.We included inpatients diagnosed with schizophrenia between 1 January 2022, and 31 December 2024. Patients aged ≥18 years, with good medication adherence, and hospitalized for 6 months or more were included. Based on whether the study subjects used TCM during hospitalization, they were divided into an exposed group and a non-exposed group, and the outcomes of both groups were retrospectively tracked, specifically the occurrence of MetS. We first used univariate analysis to screen for potential confounding variables. Then, multivariate logistic regression analysis was performed to adjust for the influence of confounding factors; finally, the impact of exposure factors on the study outcomes was assessed, and odds ratios (OR) were calculated.

**Results:**

A total of 897 patients were included in this study. The average age was 47.68 years ± 14.67 years. Among them, 163 patients (18.17%) were in the TCM exposed group, and 247 patients (27.53%) had MetS. The incidence of MetS in the TCM exposed group was 17.18%, while the incidence in the non-exposed group was 29.84%. The incidence of “central obesity” (29.5% vs. 39.8%) and “hyperglycemia” (13.50% vs. 21.8%) in the TCM exposed group was lower than that in the non-exposed group respectively. Binary logistic regression results showed that educational background, TCM, quetiapine, clozapine, risperidone, aripiprazole, and BMI were independently associated with the occurrence of MetS. Compared to patients with lower educational levels (≤9 years), those with higher education (>12 years) had a reduced risk of developing MetS (OR: 0.45, 95% CI: 0.25–0.81, p < 0.01). Compared to patients not using TCM, medium-to long-term (≥6 months) use of TCM reduced the risk of MetS (OR: 0.50, 95% CI: 0.30–0.83, p < 0.01). Patients using risperidone (OR: 0.54, 95% CI: 0.36–0.83, p < 0.01) and aripiprazole (OR: 0.39, 95% CI: 0.21–0.72, p < 0.01) had relatively lower risks of MetS incidence compared to patients taking any other antipsychotic drugs. The incidence of MetS showed varying degrees of reduction. A higher BMI is positively correlated with an increased risk of MetS (OR: 1.39, 95% CI: 1.32–1.46, p < 0.001). Compared to patients not using quetiapine or clozapine, those who used quetiapine (OR: 1.86, 95% CI: 1.11–3.13, p < 0.05) or clozapine (OR: 1.74, 95% CI: 1.14 - 2.68, p < 0.05) had an increased risk of developing MetS.

**Conclusion:**

In this retrospective cohort study, the occurrence of MetS among hospitalized patients with schizophrenia was common. Exposure to quetiapine, clozapine and increased BMI were significant risk factors for MetS; while exposure to TCM, aripiprazole, risperidone, and having higher education were protective factors against the occurrence of MetS. It is necessary to detect and prevent MetS in patients with schizophrenia. Long-term TCM treatment can reduce the incidence of MetS, providing a better alternative and direction for patients with chronic schizophrenia.

## 1 Introduction

Schizophrenia is a chronic and disabling mental disorder that affects approximately 1% of the global population. To control symptoms and prevent relapse, patients require long-term treatment with antipsychotic medications (Jauhar et al., 2022; Moilanen et al., 2015). Second-generation antipsychotics (SGAs) such as olanzapine and clozapine have clinical efficacy, but long-term use of these medications is closely associated with metabolic disorders, including weight gain, dyslipidemia.demia, and insulin resistance (Tschoner et al., 2009; Rojo et al., 2015). These adverse reactions increase the risk of MetS in schizophrenia patients by 2–3 times compared to the general population, significantly raising the incidence and mortality of cardiovascular diseases (Joshi et al., 2013; Burghardt and Ellingrod, 2013; Shojaeimotlagh et al., 2019).

The definition of MetS includes central obesity, hypertension, hyperglycemia, and dyslipidemia, with 19%–52% (Saari et al., 2005; Subashini et al., 2011; Correll et al., 2010) of schizophrenia patients receiving antipsychotic medication suffering from this condition. Current strategies to alleviate MetS include lifestyle interventions, pharmacological aids (such as metformin), and switching antipsychotic medications, but these strategies face numerous challenges, such as poor patient compliance and limited efficacy (De Boer et al., 2021). Therefore, there is an urgent need to adopt a complementary approach to address metabolic disorders without affecting the mental stability of the individual’s state.

Traditional Chinese Medicine (TCM) has garnered attention for its potential to regulate metabolic pathways through multi-target mechanisms (Liu et al., 2016; Chen et al., 2024; Dang et al., 2025). Preclinical and clinical observations suggest that TCM herbal formulations may improve glucose homeostasis, lipid metabolism, and inflammatory markers through pathways involving AMPK activation, gut microbiota regulation, and enhanced mitochondrial function (Meng et al., 2020; Wu et al., 2022). Notably, herbs such as Bupleurum chinense (Huanglian Wendan Decoction) and Coptis chinensis (Diankuang Mengxing Decoction) exhibit dual neuroprotective and metabolic regulatory properties (Guo et al., 2022; Hu et al., 2018; Lei et al., 2024; Tian E. et al., 2023).

However, there are significant gaps in the existing research: Firstly, current research on TCM in schizophrenia primarily focuses on alleviating acute symptoms (Che et al., 2016; Rathbone et al., 2007), but there is a lack of reliable longitudinal assessments of the long-term effects on metabolic outcomes, and especially a lack of real-world research evidence (Pan and Kong, 2018; Wu et al., 2020; Zhao et al., 2024). Secondly, the synergistic mechanisms between traditional Chinese medicine and antipsychotic drugs (such as oxidative stress, hypothalamic-pituitary-adrenal axis regulation, adipokine secretion) are not clearly defined, and the impact of unmeasured confounders in the real world on the results has not been adequately considered.This knowledge gap is crucial because the chronic nature of schizophrenia requires interventions that can simultaneously address both mental and metabolic health issues over a long period (Jones and Jones, 2008; Petrova and Semenova, 2024).

To address the knowledge gap in long-term metabolic effects of TCM as an adjunct to antipsychotic therapy in schizophrenia, this study uses a real-world retrospective cohort design (6–36 months follow-up, large clinical dataset) to evaluate how specific classic TCM formulas—Huanglian Wendan Decoctionand and Diankuang Mengxing Decoction (incorporating herbs with dual neuroprotective/metabolic benefits)—impact MetS incidence when combined with antipsychotics. We analyze influencing factors (antipsychotic type, baseline metabolism) and, using real-world data (vs. idealized RCTs), test if these formulas can delay/reverse antipsychotic-induced metabolic decline while preserving mental stability. The innovation lies in focusing on classic formula-based interventions (not isolated herbs) to address neglected long-term metabolic outcomes in TCM-antipsychotic combinations, answering: Can these defined formulas mitigate metabolic deterioration via multi-target mechanisms over time?

## 2 Materials and methods

### 2.1 Clinical data

#### 2.1.1 Study population

The study population consisted of hospitalized patients diagnosed with schizophrenia at Xiamen Xianyue Hospital from 1 January 2022, to 31 December 2024. The sample size estimation was based on an expected prevalence of MetS of 46% (Ellingrod et al., 2012) in the non-TCM exposure group and the relative risk (RR) is 1.5 (Jiang et al., 2019). The significance level was set at 0.05, and the confidence level was set at 90%. The calculated minimum required sample size of TCM exposure group is 122, while that of non-TCM exposure group is 545. (non-exposed group: exposed group = 4.5).

#### 2.1.2 Inclusion criteria

1. Patients with schizophrenia should meet the diagnostic criteria for schizophrenia in the International Classification of Diseases (ICD-10);
2. Age ≥ 18 years;
3. Good medication adherence (Medication coverage rate≥80%);
4. Hospitalization duration of 6 months or more.

#### 2.1.3 Exclusion criteria

**FIGURE 1:**
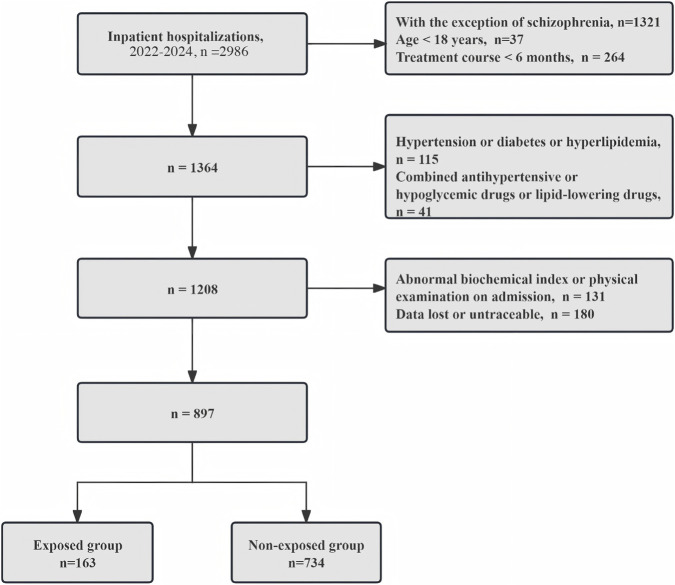
The case screening process for the TCM exposed group and non-exposed group.

1. Abnormal indicators at admission, such as diastolic blood pressure (DBP), systolic blood pressure (SBP), and laboratory tests like fasting blood sugar (FBS), triglycerides (TG), cholesterol (TC), high-density lipoprotein cholesterol (HDL-C), and low-density lipoprotein cholesterol (LDL-C);
2. Diagnosis of accompanying organic diseases at admission, such as hypertension, diabetes, and hyperlipidemia;
3. Presence of combined medication orders for comprehensive departments at admission, such as antihypertensives, antidiabetics, and lipid-lowering drugs;
4. Missing or untraceable laboratory and physical examination data during admission and discharge. The detailed process is shown in Figure 1.

A total of 897 patients were included, with 163 in the exposed group and 734 in the non-exposed group.

### 2.2 Study design

This study employed a retrospective cohort study design. By collecting, screening, and analyzing the historical medical records of patients with schizophrenia, the association between the treatment of TCM combined with antipsychotic drugs and the prevalence of MetS was explored. The subjects were divided into exposed and non-exposed groups based on whether they used TCM during hospitalization, and the occurrence of study outcomes, namely, the incidence of MetS, was retrospectively tracked in both groups. The follow-up period was ≥ 6 months, from the time of patient admission until the occurrence of MetS events or the study end date (31 December 2024). The median follow-up time was 17 months (ranging from 6 to 36 months).

### 2.3 Data sources

The research data comes from the hospital’s case management system, including patients’ basic demographic information, medical history, physical examinations, laboratory tests, patient diagnoses, and medication treatment details. Demographic information includes age, gender, employment status, marital status, and education level. Physical examinations include weight, height, BMI, waist circumference (WC), diastolic blood pressure (DBP), and systolic blood pressure (SBP). Laboratory tests include fasting blood sugar (FBS), triglycerides (TG), cholesterol (TC), high-density lipoprotein cholesterol (HDL-C), and low-density lipoprotein cholesterol (LDL-C). Medication treatment details include types of antipsychotics, concomitant medications, use of antiepileptic drugs, use of antidepressants, use of sleeping pills, and use of traditional Chinese medicine. All data were uniformly extracted by trained researchers and verified through double-checking to ensure accuracy.

### 2.4 Explanation of covariates

1. Mood stabilizers include antiepileptic drugs and lithium salts, excluding antipsychotic medications. Antiepileptic drugs include sodium valproate, carbamazepine, lamotrigine, topiramate, and levetiracetam. The usage rate of antiepileptic drugs such as valproic acid is relatively low. The single-factor analysis shows no statistical difference. Therefore, it is presented in a binary classification form of “whether to use mood stabilizers”.
2. Antidepressants include fluoxetine, paroxetine, sertraline, fluvoxamine, citalopram, venlafaxine, duloxetine, mirtazapine, clomipramine, and bupropion. The usage rate of antidepressants is low, so the binary classification variable “whether to use antidepressants” was included in the regression model.
3. Sleeping pills include benzodiazepines (BZDs) and non-benzodiazepines (Non-BZDs), excluding antidepressants and antipsychotics with sedative effects.
4. Age categories are as follows: youth (18–39 years), middle-aged (40–64 years), and elderly (65 years or older).

### 2.5 Treatment methods

Antipsychotic treatment in the experimental group (Chinese herbal medicine combined with antipsychotic medication) and the control group (antipsychotic medication only): Follow the principles of drug treatment in the Chinese guidelines for the prevention and treatment of schizophrenia (Lu and Yu, 2020).

#### 2.5.1 The diagnostic criteria for the “DianKuang” syndrome

The syndrome differentiation was independently completed by two senior or higher-level Chinese medicine practitioners (all with ≥10 years of clinical experience in Chinese medicine psychiatry).

1. “Dian” syndrome (belongs to Yin, deficiency of multiple types): mental depression, silence and dementia, murmuring to oneself, quiet and less movement, with a cheerful expression (manifested as negative symptoms of schizophrenia: emotional flatness, reduced will, poverty of speech).
2. “Kuang” syndrome (belongs to Yang, excess of multiple types): manic outbursts and scolding; disturbance and unrest; easily angered when in motion (manifested as positive symptoms of schizophrenia: hallucinations and delusions, excitement and impulsivity, disordered thinking).
3. Mixed symptoms syndrome: The above two types of symptoms overlap.

#### 2.5.2 Chinese medicine treatment in the experimental group

Based on the aforementioned drug treatment, The herbal treatment in the experimental group meets the diagnostic and therapeutic principles of “DianKuang” syndrome in Traditional Chinese Medicine.

1. “Dian” syndrome

Treatment method: Regulate qi and resolve phlegm, harmonize the stomach and benefit the gallbladder. Formula: Huanglian Wendan Decoction (from “Liu Yin Tiao Bian”) with modifications. The formula includes coptis, Pinellia, dried tangerine peel, Poria, roasted licorice, South China star anise, bitter orange, bamboo shavings, sour jujube seed, distant wisdom herbs, gardenia, gentian, jujube, and others. The core ingredients of Huanglian Wendan Decoction are coptis, pinellia, dried tangerine peel, poria, bamboo shavings, and licorice, which account for more than 70% of the total medicinal ingredients to ensure the standardization and reproducibility of the formula. The dosage range for the core ingredients is coptis, 3–6 g, pinellia 6–10 g, dried tangerine peel 9–12 g,poria 15–20 g, and bamboo shavings 9–12 g. The medication frequency is 1 dose per day, taken orally in two divided doses after decoction.

2. “Kuang” syndrome

Treatment method: Regulate qi to relieve depression, dispel stasis, and open the orifices. Formula: Diankuang Mengxing Decoction (from “Yilin Gai Cuo”) with modifications. The formula includes peach kernel, red peony root, bupleuri radix, cyperus, akebia, pinellia, dried tangerine peel, green tangerine peel, perilla seed, and licorice among others. The core medicinal ingredients of Diankuang Mengxing Decoction are peach kernel, red peony root, bupleuri radix, cyperus, pinellia, dried tangerine peel, and licorice, accounting for over 80%, ensuring the formula can be replicated. The dosage range for the core medicines is: peach kernel 9–12 g, red peony root 12–15 g, bupleuri radix 9–12 g, cyperus 9–12 g, pinellia 6–10 g. Frequency: 1 dose per day, decocted in water and taken orally in 2 doses.

3. Mixed Real and Illusion Syndrome: Combine the two formulas and make adjustments (double the dosage of the main symptom formula, supplemented with drugs for regulating the spleen and stomach).

### 2.6 Diagnostic criteria for metabolic syndrome

The diagnostic criteria for metabolic syndrome in patients with schizophrenia refer to the new definition of metabolic syndrome in the 2017 Chinese Guidelines for the Prevention and Treatment of Type 2 Diabetes (Chinese Diabetes Society, 2018). A diagnosis can be made if three or more of the following criteria are met:

1. Abdominal obesity (i.e., central obesity): waist circumference ≥90 cm for men and ≥85 cm for women;
2. Hyperglycemia: fasting blood glucose ≥6.1 mmol/L or 2-h post-glucose load blood glucose ≥7.8 mmol/L and/or diagnosed diabetes with treatment;
3. Hypertension: blood pressure ≥130/85 mmHg and/or confirmed hypertension with treatment;
4. Fasting triglycerides (TG) ≥1.70 mmol/L;
5. Fasting high-density lipoprotein cholesterol (HDL-C) <1.04 mmol/L.

### 2.7 Statistical analysis

Continuous variables are expressed as mean ± standard deviation (x ± s), and intergroup comparisons are performed using t-tests; categorical variables are expressed as frequency [n (%)], and intergroup comparisons are conducted using chi-square tests or Fisher’s exact tests. Descriptive statistical analysis is used for patients’ demographic data, medication treatment characteristics, medical history, etc. Univariate analysis is first used to screen for potential confounding variables (p value < 0.10); then multivariate logistic regression analysis is employed to adjust for the influence of confounding factors; finally, the impact of exposure factors on study outcomes is assessed, calculating odds ratios (OR) and their 95% confidence intervals (95% CI). Model validation and AUC estimation: Due to the limited data volume of the retrospective study, no independent test set was divided. Instead, 10-fold cross-validation combined with DeLong test was adopted. Data processing and analysis are performed using SPSS 20.0 statistical software, with p < 0.05 indicating statistical significance.

### 2.8 Ethical approval

This study was approved by the Medical Ethics Committee of Xiamen Xianyue Hospital and was exempt from informed consent. As this study is retrospective, patients were not subjected to any interventions, and there was no involvement of patient privacy disclosure.

## 3 Results

### 3.1 Comparison of baseline general data between two groups

In this study, a total of 897 patients with schizophrenia were included, with males accounting for 58.97% and females for 41.03%, and the average age was 47.68 ± 14.67 years. Among them, there were 163 cases in the TCM exposure group and 734 cases in the non-exposure group. Among the 163 cases in the exposed group, there were 42 cases of “Dian” syndrome, 68 cases of “Kuang” syndrome, and 53 cases of mixed syndrome.The differences in BMI (t = 1.880, p = 0.061) and duration of illness (t = 1.647, p = 0.100) between the two groups were not significant; however, there were significant differences in age (χ^2^ = 8.926, p = 0.012), gender (χ^2^ = 49.263, p < 0.001), marital status (χ^2^ = 13.054, p = 0.001), drinking (χ^2^ = 16.282, p < 0.001), and age of onset (t = 2.512, p = 0.012), as detailed in Table 1.

**TABLE 1:** Comparison of baseline characteristics between cases (TCM Exposure) and controls (Not TCM Exposure).

Characteristics		No. (%) of patients		*P*
		TCM exposure (n = 163)	Not TCM exposure (n = 734)	
Age of onset, mean ± SD		25.37 ± 9.73	27.69 ± 10.85	0.012
Disease course, mean ± SD		18.88 ± 12.42	20.79 ± 13.60	0.1
BMI, mean ± SD		23.28 ± 3.71	23.90 ± 4.34	0.061
Age group, years		​	​	0.012
​	Youth (18–39)	65 (39.9)	214 (29.2)	​
	Middle age (40–64)	82 (50.3)	426 (58.0)	​
	Elderly (≥65)	16 (9.8)	94 (12.8)	​
Sex		​	​	<0.001
​	Female	27 (16.6)	341 (46.5)	​
	Male	136 (83.4)	393 (53.5)	​
Nation		​	​	​
​	The Han nationality	162 (99.4)	727 (99.0)	1
	Others	1 (0.6)	7 (1.0)	​
Marital status		​	​	​
​	Unmarried	113 (69.3)	398 (54.2)	0.001
	Get married	31 (19.0)	185 (25.2)	​
	Divorced or widowed	19 (11.7)	151 (20.6)	​
Place of residence		​	​	0.275
​	Rural areas	74 (45.4)	299 (40.7)	​
	Urban area	89 (54.6)	435 (59.3)	​
Education level, years		​	​	0.151
​	≤9	32 (19.6)	198 (27.0)	​
	9–12	98 (60.1)	402 (54.8)	​
	>12	33 (20.3)	134 (18.2)	​
Job		​	​	0.091
​	Yes	16 (9.8)	45 (6.1)	​
	No	147 (90.2)	689 (93.9)	​
Drinking		​	​	<0.001
​	Yes	22 (13.5)	36 (4.9)	​
	No	141 (86.5)	698 (95.1)	​
Smoking		​	​	0.069
​	Yes	50 (30.7)	175 (23.8)	​
	No	113 (69.3)	559 (76.2)	​
History of liver disease		​	​	0.178
​	Yes	15 (9.2)	46 (6.3)	​
	No	148 (90.8)	688 (93.7)	​
Allergic history		​	​	0.94
​	Yes	8 (4.9)	35 (4.8)	​
	No	155 (95.1)	699 (95.2)	​
History of kidney disease		​	​	1
​	Yes	2 (1.2)	7 (1.0)	​
	No	161 (98.8)	727 (99.0)	​

### 3.2 Comparison of MetS incidence between TCM exposure and non-exposure group

Among 897 patients with schizophrenia, 247 cases of MetS were reported, resulting in an incidence rate of 27.53%. The incidence rate of MetS in the TCM exposure group was 17.18%, while in the non-exposure group it was 29.84%, showing a significant difference between the two groups. The TCM exposure group had lower incidence rates for “central obesity” and “hyperglycemia,” at 29.45% and 13.50%, respectively. Without considering the influence of confounding factors, long-term TCM treatment seems to reduce the incidence of MetS in patients with schizophrenia, with an OR value of 0.49, as detailed in Table 2.

**TABLE 2:** Comparison of MetS incidence between cases (TCM Exposure) and controls (Not TCM Exposure).

MetS indicators	No. (%) of patients		Crude 0R (95% CI)	*P*
	TCM exposure (n = 163)	Not TCM exposure (n = 734)		
Central obesity	48 (29.5)	292 (39.8)	0.63 (0.44–0.91)	0.014
Hyperglycemia	22 (13.5)	160 (21.8)	0.56 (0.35–0.91)	0.017
Hypertension	43 (26.4)	187 (25.5)	1.05 (0.71–1.54)	0.811
Fasting TG ≥ 1.70 mmol/L	65 (39.9)	325 (44.3)	0.84 (0.59–1.18)	0.305
Fasting HDL-C<1.04 mmol/L	48 (29.5)	257 (35.0)	0.78 (0.54–1.12)	0.176
MetS	28 (17.2)	219 (29.8)	0.49 (0.32–0.76)	0.001

### 3.3 Univariate analysis of MetS incidence

Considering the potential impact of relevant confounding factors on the occurrence of MetS, this study included 35 covariates, including general demographic data, medical history, and medication treatment. The results of the univariate analysis of MetS incidence showed significant differences in 13 covariates between the two groups: age group (χ^2^ = 14.159, p < 0.001), marital status (χ^2^ = 8.518, p = 0.014), educational background (χ^2^ = 11.231, p = 0.004), jop (χ^2^ = 4.073, p = 0.044), TCM (χ^2^ = 10.711, p = 0.001), emotional stabilizers (χ^2^ = 3.872, p = 0.049), sleeping pills (χ^2^ = 6.160, p = 0.013), quetiapine (χ^2^ = 8.779, p = 0.003), clozapine (χ^2^ = 8.551, p = 0.003), risperidone (χ^2^ = 5.499, p = 0.019), aripiprazole (χ^2^ = 11.432, p < 0.001), BMI (t = −16.560, p = 0.000), and disease duration (t = −3.528, p = 0.000). For details, see Table 3.

**TABLE 3:** Univariate analysis of MetS incidence.

Variables		No. (%) of patients		*P*
		MetS (n = 247)	Not MetS (n = 650)	
Age of onset, mean ± SD		27.10 ± 10.74	27.33 ± 10.67	0.776
Disease course, mean ± SD		22.99 ± 12.97	19.47 ± 13.46	<0.001
BMI, mean ± SD		27.12 ± 3.48	22.52 ± 3.79	0.000
Age group, years		​	​	<0.001
​	Youth (18–39)	61 (24.7)	218 (33.5)	​
	Middle age (40–64)	145 (58.7)	367 (56.5)	​
	Elderly (≥65)	41 (16.6)	65 (10)	​
Sex		​	​	0.265
​	Female	153 (61.9)	376 (57.9)	​
	Male	94 (38.1)	274 (42.2)	​
Nation		​	​	1.000
​	The Han nationality	245 (99.2)	644 (99.1)	​
	Others	2 (0.8)	6 (0.9)	​
Marital status		​	​	0.014
​	Unmarried	132 (53.4)	379 (58.3)	​
	Get married	53 (21.5)	163 (25.1)	​
	Divorced or widowed	62 (25.1)	108 (16.6)	​
Place of residence		​	​	0.141
​	Rural areas	93 (37.7)	280 (43.1)	​
	Urban area	154 (62.4)	370 (56.9)	​
Education level, years		​	​	0.004
​	≤9	73 (29.6)	157 (24.2)	​
	9–12	145 (58.7)	355 (54.6)	​
	>12	29 (11.7)	138 (21.2)	​
Job		10 (4.1)	51 (7.9)	0.044
Drinking		17 (6.9)	41 (6.3)	0.754
Smoking		71 (28.7)	154 (23.7)	0.119
Allergic history		11 (4.5)	32 (4.9)	0.769
History of liver disease		14 (5.7)	47 (7.2)	0.406
History of kidney disease		2 (0.8)	7 (1.1)	1.000
TCM		28 (11.3)	135 (20.8)	0.001
Drug combination		191 (77.3)	514 (79.1)	0.568
Antidepressant		16 (6.5)	54 (8.3)	0.361
Mood stabilizer		61 (24.7)	122 (18.8)	0.049
Sleeping pill		85 (34.4)	283 (43.5)	0.013
Olanzapine		94 (38.1)	250 (38.5)	0.911
Quetiapine		41 (16.6)	62 (9.5)	0.003
Clozapine		64 (25.9)	112 (17.2)	0.003
Risperidone		54 (21.9)	193 (29.7)	0.019
Paliperidone		20 (8.1)	67 (10.3)	0.318
Aripiprazole		21 (8.5)	114 (17.5)	<0.001
Amisulpride		27 (10.9)	87 (13.4)	0.324
Perospirone		7 (2.8)	19 (2.9)	0.943
Blonanserin		6 (2.4)	16 (2.5)	0.978
Chlorpromazine		7 (2.8)	10 (1.5)	0.319
Haloperidol		4 (1.6)	3 (0.5)	0.182
Perphenazine		4 (1.6)	2 (0.3)	0.052
Sulpiride		2 (0.8)	7 (1.1)	1.000
Ziprasidone		0 (0.0)	3 (0.5)	0.565
Lurasidone		5 (2.0)	16 (2.5)	0.699

### 3.4 Multivariate binary logistic regression analysis of MetS incidence

Model comparison shows that the AUC of Model 1 and Model 2 are 0.857 (95% CI: 0.833–0.880) and 0.851 (95% CI: 0.826–0.873), respectively. The DeLong test confirmed that there is no significant difference in predictive performance between the two (Z = 0.31, p = 0.76), suggesting equivalent ability to distinguish the risk of metabolic syndrome (MetS). Model 2 was chosen as the final model based on three considerations: first, the performance is comparable under statistical equivalence, meeting the predictive needs; second, clinical completeness—Model 2 includes clozapine, which is known to significantly increase MetS risk, thus more comprehensively reflecting the key factor of “high-risk drug exposure”; third, alignment with research objectives—when assessing the impact of traditional Chinese medicine combined treatment on MetS, it is necessary to control for known metabolic risk factors, including clozapine. By incorporating this drug, Model 2 can more accurately quantify the independent effects of traditional Chinese medicine intervention and baseline metabolic status, with its OR value being closer to the net impact in real clinical scenarios. In summary, although Model 1 has a slightly higher predictive value, Model 2 was selected as the final model due to its equivalence, completeness, and research adaptability, with its OR value better reflecting the actual impact of key risk factors.

The results of Model 2 show that educational background, TCM, quetiapine, clozapine, risperidone, aripiprazole,and BMI are independently associated with the incidence of MetS. Compared to patients with illiteracy or primary education, those with higher education have a lower risk of developing MetS (OR: 0.45, 95% CI: 0.25–0.81, p < 0.01). Compared to patients not using TCM, those who used TCM for a medium to long term had a decreased risk of developing MetS (OR: 0.50, 95% CI: 0.30–0.83, p < 0.01). Patients using risperidone (OR: 0.54, 95% CI: 0.36–0.83, p < 0.01) and aripiprazole (OR: 0.39, 95% CI: 0.21–0.72, p < 0.01) showed varying degrees of lower incidence of MetS compared to those not using these medications. For details, see Table 4.

**TABLE 4:** Multivariate binary logistic regression analysis of MetS incidence.

Variables		Model 1		Model 2	
		OR (95% CI)	*P*	OR (95% CI)	*P*
Age group, years					
​	Youth (18–39)	Reference	0.181	——	——
	Middle age (40–64)	1.31 (0.78–2.21)	0.309	——	——
	Elderly (≥65)	2.11 (0.94–4.72)	0.070	——	——
Marital status					
​	Unmarried	Reference	0.320	——	——
	Get married	0.78 (0.49–1.24)	0.296	——	——
	Divorced or widowed	1.18 (0.73–1.90)	0.511	——	——
Education level, years					
​	≤9	Reference	0.069	Reference	0.020
	9–12	0.98 (0.63–1.50)	0.911	0.90 (0.60–1.34)	0.592
	>12	0.52 (0.28–0.97)	0.041	0.45 (0.25–0.81)	0.007
Job		0.80 (0.35–1.81)	0.592	——	——
TCM		0.55 (0.33–0.92)	0.022	0.50 (0.30–0.83)	0.007
Mood stabilizer		0.98 (0.63–1.54)	0.940	——	——
Sleeping pill		0.87 (0.60–1.28)	0.481	——	——
Quetiapine		1.89 (1.10–3.22)	0.020	1.86 (1.11–3.13)	0.019
Clozapine		1.54 (0.98–2.40)	0.059	1.74 (1.14–2.68)	0.011
Risperidone		0.57 (0.37–0.87)	0.010	0.54 (0.36–0.83)	0.004
Aripiprazole		0.42 (0.23–0.78)	0.006	0.39 (0.21–0.72)	0.003
Perphenazine		4.53 (0.44–47.01)	0.206	——	——
BMI value		1.40 (1.32–1.48)	0.000	1.39 (1.32–1.46)	<0.001
Disease course		1.01 (0.99–1.02)	0.588	——	——

Model 1: Fully ajusted model: Variables that met the criterion of P < 0.10 in all single-factor analyses (Age group, marital status, Education level, Job, TCM, mood stabilizer, Sleeping pill, Quetiapine, Clozapine, Risperidone, Aripiprazole, Perphenazine, BMI value, Disease course).

Model 2: Adjusted for education level,TCM, quetiapine, clozapine, risperidone, aripiprazole and BMI value.

The risperidone reference group consists of patients who did not use risperidone (including those using high-risk drugs such as chlorpromazine), reflecting the relative effect rather than absolute protection. Due to the inclusion of high-risk drugs in the reference group, interpretation should be made with caution in light of the clinical context.

A higher BMI is positively correlated with an increased risk of MetS (OR: 1.39, 95% CI: 1.32–1.46, p < 0.001). Compared to patients not using quetiapine, those who used quetiapine had an elevated risk of developing MetS (OR: 1.86, 95% CI: 1.11–3.13, p < 0.05). In addition, clozapine was also a risk factor for the occurrence of MetS (OR: 1.74, 95% CI: 1.14 - 2.68, p < 0.05). For details, see Table 4.

### 3.5 Multivariate binary logistic regression analysis of MetS incidence by age group

To explore the differences in the influencing factors of metabolic syndrome (MetS) among schizophrenia patients of different age groups, this study conducted a binary logistic regression analysis stratified by age (young: 18–39 years, middle-aged: 40–64 years, elderly: ≥65 years). It should be noted that the sample sizes in each age group are relatively small, and the results may be affected by sampling bias. Therefore, the following analysis should be considered as exploratory findings and interpreted with caution, and future studies with larger sample sizes are needed for validation.

In the middle-aged group (40–64 years) and the elderly group (≥65 years), some influencing factors preliminarily suggest a protective effect (Table 5): TCM treatment reduces the risk of MetS in the middle-aged group (OR = 0.47, 95% CI: 0.24–0.94, P < 0.05), and the protective effect is more significant in the elderly group (OR = 0.13, 95% CI: 0.02–0.83, P < 0.05), indicating that Chinese medicine may have potential regulatory value for metabolic disorders in middle-aged and elderly patients; risperidone treatment reduces the risk of MetS in the middle-aged group (OR = 0.51, 95% CI: 0.30–0.88, P < 0.05) and the elderly group (OR = 0.22, 95% CI: 0.06–0.78, P < 0.05), which needs to be further explored in conjunction with baseline characteristics.

**TABLE 5:** Multivariate binary Logistic Regression Analysis of MetS Incidence by Age Group.

Variables		Youth (18–39) n = 279		Middle age (40–64) n = 508		Elderly (≥65) n = 110	
		Adjusted OR (95% CI)	*P*	Adjusted OR (95% CI)	*P*	Adjusted OR (95% CI)	*P*
Education level, years							
​	≤9	Reference	0.023	——	——	——	——
	9–12	0.34 (0.09–1.31)	0.116	——	——	——	——
	≥12	0.13 (0.03–0.59)	0.009	——	——	——	——
Smoking		2.63 (1.10–6.28)	0.030	——	——	——	——
TCM		——	——	0.47 (0.24–0.94)	0.032	0.13 (0.02–0.83)	0.031
Quetiapine		——	——	2.25 (1.16–4.37)	0.016	——	——
Clozapine		——	——	2.15 (1.24–3.74)	0.006	——	——
Risperidone		——	——	0.51 (0.30–0.88)	0.015	0.22 (0.06–0.78)	0.019
Aripiprazole		0.09 (0.02–0.37)	<0.001	——	——	——	——
Paliperidone		——	——	0.34 (0.12–0.95)	0.042	——	——
Perphenazine		——	——	16.34 (1.07–248.46)	0.044	——	——
BMI value		1.57 (1.38–1.78)	<0.001	1.39 (1.30–1.50)	<0.001	1.47 (1.24–1.75)	<0.001

In the young group (18–39 years), higher education (≥12 years) (OR = 0.13, 95% CI: 0.03–0.59, P < 0.01) and aripiprazole treatment (OR = 0.09, 95% CI: 0.02–0.37, P < 0.001) are potential protective factors, with the former suggesting that education level may indirectly influence metabolic status through health cognition, and the latter consistent with the consensus on aripiprazole as a “low metabolic risk SGA.” (Table 5).

## 4 Discussion

Our research shows that the prevalence of MetS among hospitalized patients with schizophrenia (27.53%) is significantly higher than that of the general population or healthy controls. This result differs considerably from previous studies, which reported the prevalence of MetS in schizophrenia patients to be between 19% and 52% (Correll et al., 2010; Saari et al., 2005; Subashini et al., 2011). For example, in a cross-sectional study by Saloojee S et al. on the prevalence of MetS and associated risk factors in severe mental illness, the prevalence of MetS in schizophrenia was observed to be 20.8% (Saloojee et al., 2016). This value is lower than that in our study, which may be due to their study population being predominantly Black and White, and the participants being relatively younger (aged 18–65 years). Another study on the genetic effects of folic acid medication on MetS found that 46% of schizophrenia patients had MetS (Ellingrod et al., 2012), which is much higher than our study’s value. However, it should be noted that in our study, 18.17% of cases had been receiving long-term treatment with both Western and traditional Chinese medicine. Additionally, the inclusion and exclusion criteria for cases may differ due to varying research objectives. Different treatment methods and case selection criteria could also lead to discrepancies in the prevalence of MetS. In summary, the differences in MetS prevalence may be attributed to inconsistencies in the study population, experimental design, treatment methods, and observation periods.

Compared to the nearly equal male-to-female distribution in general hospitals, we found that among the included hospitalized patients with schizophrenia, males accounted for 58.97%, with a male-to-female ratio of 1.44:1. This is generally consistent with previous epidemiological studies on schizophrenia (Barton et al., 2020; Sommer et al., 2020). There may be two reasons for this difference. First, estrogen may have a neuroprotective effect (Wu et al., 2013), leading to a delayed onset in female patients. Second, poorer pre-morbid adaptation in males may also contribute to this difference (Abel et al., 2010).

Consistent with previous studies (Krijnen et al., 2022; Reyes-Ortiz et al., 2024), our findings indicate that education level is negatively correlated with the development of metabolic syndrome (MetS), particularly that higher education serves as a protective factor against MetS. Specifically, a cohort study on the impact of educational duration on MetS showed that individuals with lower education levels typically develop MetS 2.3 years earlier and spend an additional 2.6 years living with MetS compared to those with higher education (Hoveling et al., 2023). Another study on educational disparities in MetS mentioned that different levels of education create socioeconomic differences that may increase the risk of developing MetS (Kim et al., 2018).

Few published studies provide directly comparable results. Previous research has only been comparatively limited to a certain extent. Our study shows that long-term use of TCM treatment is a protective factor for the prevalence of MetS in hospitalized patients with schizophrenia, particularly in controlling weight and blood sugar levels. This result is similar to previous studies (Huang et al., 2015; Shi et al., 2022; Tian Y. et al., 2023; Zhou et al., 2023). A study on the mechanism of traditional Chinese medicine formulas in treating metabolic syndrome indicates that the main pharmacological action of Wen Dan Decoction in improving metabolic syndrome lies in maintaining lipid and glucose metabolism, anti-cancer activity, as well as immune regulation and liver protection (Chen et al., 2016). Among the ten components of Wen Dan Decoction, hesperidin, quercetin, naringin, and tangeretin may play a more significant role in regulating hyperlipidemia (Xu et al., 2024). At the molecular mechanism level, Wen Dan Decoction may treat metabolic disorders by regulating PPARγ/NF-κB signaling and the level of ABCA1 in serum (Chen et al., 2018; Liu et al., 2025).

There is very little high-quality literature directly studying the effects of “Dian Kuang Meng Xing Tang” on metabolic syndrome in schizophrenia, with existing research mostly consisting of small samples or animal experiments. A meta-analysis (Lam et al., 2024) indicates that the combination of Dian Kuang Meng Xing Tang with different antipsychotic medications can improve the overall prognosis of schizophrenia patients and reduces the occurrence of adverse reactions. Additionally, another clinical study (Pan et al., 2021) found that the combination of Dian Kuang Meng Xing Tang with aripiprazole significantly improved the symptoms of schizophrenia with liver qi stagnation and phlegm obstruction, enhancing the patients’ lipid metabolism and suppressing inflammatory responses. TCM treatment for MetS has unique advantages due to its holistic concept and multi-target regulation.

Consistent with previous studies (Kahl et al., 2013; Paredes et al., 2014; Ventriglio et al., 2018), our findings indicate that exposure to quetiapine (OR: 1.885, 95% CI: 1.104–3.216) or clozapine (OR: 1.74, 95% CI: 1.14–2.68) is a risk factor for MetS, with its OR value significantly higher than that of other antipsychotic medications. Notably, a randomized, double-blind, placebo-controlled study (Tocco et al., 2021) showed that compared to placebo, quetiapine increased the prevalence of MetS by 3.49 times (OR: 3.4, 95% CI: 1.93–6.29), which is much higher than our study results. However, it is important to note that this study used a placebo as the control group, while our study used patients on other antipsychotic medications besides quetiapine as the control group. Additionally, a study integrating data from 32 psychiatric hospitals in China, using ziprasidone (an antipsychotic with low metabolic syndrome risk) as a reference, reached similar conclusions, indicating that the risk of metabolic syndrome associated with quetiapine increased to (OR = 3.29, P <0 .001) (Zhang et al., 2020). Therefore, the differences in the choice of reference subjects in experimental studies often lead to significant variations in the risk of MetS occurrence. There are two reasons for the high prevalence of MetS caused by quetiapine and clozapine. First, quetiapine and clozapine may affect neuropeptide and AMP-activated protein kinase (AMPK) activity through dopamine, serotonin, acetylcholine, and histamine receptors, resulting in hyperphysiological sympathetic outflow and increased levels of glucagon and hepatic glucose production (Carli et al., 2021). Additionally, quetiapine may reduce patients’ oxygen consumption and respiratory control rate (Scaini et al., 2018).

Unlike previous studies (Carli et al., 2021; Kahl et al., 2013; Paredes et al., 2014; Vancampfort et al., 2015; Ventriglio et al., 2018), we did not observe that olanzapine constitute risk factors for the occurrence of MetS.There are three reasons for this result. First, In model 2, it is possible to set “high-risk SGA users who did not use Olanzapine” (such as those using Clozapine and Quetiapine) as the reference group, rather than “completely not using SGA,” resulting in the “high risk” of Olanzapine being diluted by the “low risk” of the reference group. Second, most of the aforementioned studies primarily observed the prevalence of MetS with monotherapy of antipsychotic drugs (Lee et al., 2017), while our study included cases of combination therapy reaching 78.5%. Lastly, most of these studies were prospective cohort studies or randomized clinical trials (Tocco et al., 2021), often using a placebo or healthy population as the control group. In contrast, our study is a retrospective study where we utilized cases receiving other drug as the control group. The differences in research methods, drug treatment approaches, and reference subjects led to our inability to observe the risk of MetS occurrence with olanzapine.

Similar to previous studies (Carli et al., 2021; Paredes et al., 2014; Vancampfort et al., 2015; Ventriglio et al., 2018; Zhang et al., 2020), we report that aripiprazole (OR: 0.420, 95% CI: 0.225–0.784) is a protective factor against the occurrence of MetS in patients with schizophrenia. A retrospective study (Lee et al., 2011) on schizophrenia patients receiving monotherapy for MetS reached a consistent conclusion, indicating that patients treated with aripiprazole had a significantly lower likelihood of developing MetS. Research by De Hert et al. (2007) found that aripiprazole has good metabolic safety and can even revert MetS caused by other antipsychotic medications after 3 months of treatment.

Although several articles (Carli et al., 2021; Paredes et al., 2014; Ventriglio et al., 2018; Zhang et al., 2020) mention that risperidone is an antipsychotic with a moderate risk of MetS, our study concludes that risperidone (OR: 0.568, 95% CI: 0.369–0.873) is a protective factor against the occurrence of MetS in patients with chronic schizophrenia. A prospective study on metabolic syndrome in schizophrenia found that the prevalence of MetS in patients taking risperidone was 9%–24% (Saddichha et al., 2008), which is higher than the 10.82% prevalence observed in the healthy population (Tao et al., 2022). This study included cases mainly of chronic schizophrenia patients, with an average duration of 20.44 years. Due to the higher proportion of patients using antipsychotic agents with a high risk of metabolic syndrome (Olanzapine 38.3%, Clozapine 19.6%, Quetiapine 11.4%), risperidone thus becomes a relatively protective factor in this situation. Another prospective study (Wampers et al., 2012) comparing olanzapine and risperidone showed a significantly increased prevalence of MetS in the olanzapine group compared to the risperidone group, further supporting our argument.

Although the exposure to paliperidone was not included in the Model 2, the results of the logistic regression analysis of MetS across different age groups (Table 5) found that exposure to paliperidone is a potential protective factor against the occurrence of MetS. First, paliperidone is the main active metabolite of risperidone, and they share similar pharmacological mechanisms (Zhang et al., 2016). Second, compared to other second-generation antipsychotic medications (such as olanzapine and aripiprazole), paliperidone has a smaller negative impact on metabolic parameters (Schreiner et al., 2012). Paliperidone may reduce the risk of metabolic syndrome by lowering high-density lipoprotein (HDL) while having no significant effect on glucose metabolism (fasting blood glucose, HbA1c) (Zhang and Dai, 2012). In summary, paliperidone may have a relative advantage in reducing the risk of metabolic syndrome compared to other antipsychotic medications, but its potential impact on lipid metabolism should still be noted.

Consistent with previous studies (Kahl et al., 2013; Vancampfort et al., 2015; Zhang et al., 2020), our findings indicate that a higher BMI is significantly positively associated with an increased risk of MetS (OR: 1.398, 95% CI: 1.324–1.476). Although BMI is not one of the diagnostic criteria for MetS, closely monitoring the BMI of schizophrenia patients is important. Several studies exploring predictive factors for the development of MetS have shown that,BMI not only predicts the onset of MetS (Ma et al., 2024), but is also an important factor for the improvement of MetS Z-scores (Mora-Rodriguez et al., 2018). Additionally, a study on chromosomes closely related to metabolic syndrome found a strong correlation between BMI and D11S1304 (allele 1) (Daneshpour et al., 2021). However, this can only reveal correlations but cannot determine the direction of the causal relationship between BMI and MetS. Patients using drugs such as Clozapine that increase BMI are more likely to develop MetS. Medication use as a potential clinical cause may confound the association between BMI and MetS.

Previous studies have shown that the long-term treatment with ziprasidone (Paredes et al., 2014; Zhang et al., 2020) or lurasidone (Tocco et al., 2021) has the lowest probability of developing MetS. Due to the small number of cases using ziprasidone or lurasidone in the studies, we did not observe the advantage of these medications in the low prevalence of MetS. Additionally, it is possible that differences in disease diagnosis, medication treatment methods, and experimental design have contributed to our inability to observe the risk of MetS associated with mood stabilizers (Yumru et al., 2007).

In model 2, the “protective effect” of risperidone and the “non-significant risk” of olanzapine are essentially the results of real-world selection bias, reference group setting (using medium-risk SGA as a control), and the combined effects of combination therapy, rather than model failure. Through multivariate regression analysis, stratified analysis, and sensitivity analysis, we confirmed that after controlling for confounding factors such as SGA type, mood stabilizers, and baseline metabolic status, Chinese Materia Medica still reduced the risk of MetS by 50% (OR = 0.50, P < 0.01). Combining with the multi-target metabolic regulation mechanism of Traditional Chinese Medicine, we believe that the conclusion “Chinese Materia Medica is a protective factor for the prevention of MetS” is credible, providing important reference for the long-term clinical use of Chinese Materia Medica to improve the metabolic side effects of antipsychotic agents.

This study adopts a single-center design, with sample representativeness limited to a specific regional population and medical environment, namely, hospitalized patients with chronic schizophrenia. The external validity (generalizability) of the study results in other racial or cultural background populations, different medical systems (such as those in Europe and the United States), and diverse scenarios of traditional Chinese medicine practice (such as outpatient patients) needs further verification.

Despite the valuable results provided by this study, its limitations cannot be overlooked. 1. this study is a retrospective cohort study, and there are some other confounding factors, such as drug interactions, drug dosage (Ventriglio et al., 2018), and other TCM prescriptions such as those for treating colds can be used for a short period of time, that were not explored in relation to MetS. 2. The limitations of this study also include that drugs that may affect metabolism, such as Mirtazapine, Citalopram, and Valproate (with a usage rate of less than 10%), were not included in the study. Although the impact of these drugs is small, they may still introduce some bias in the results. 3. This study is based on a single-center inpatient population with chronic schizophrenia, and the representativeness of the sample is limited; the applicability of the results to community patients or other types of psychiatric populations needs further validation. 4. Furthermore, we only studied cases of hospitalization for ≥6 months, neglecting the impact of short-to medium-term traditional Chinese medicine (TCM) treatment on MetS. 5. The study relies on previous clinical data, which may be affected by inconsistencies in data collection and recording. 6. The study lacks detailed and standardized procedures for distinguishing and treating traditional Chinese medicine syndromes (“Dian” syndrome, “Kuang” syndrome), such as prescription compatibility and dose adjustment, relying on physician experience, which may affect the consistency of intervention measures and the reproducibility of results. 7. Failure to control key potential confounding factors such as diet, physical activity, and severity of psychiatric illness may lead to bias in effect estimation. Therefore, future research should focus on designing more rigorous randomized controlled trials to eliminate the influence of confounding factors and validate our findings.

## 5 Conclusion

In this retrospective cohort study, a high prevalence of MetS was observed among hospitalized patients with schizophrenia (27.53%).The exposure to clozapine, quetiapine and the increase in BMI are important risk factors for MetS, while exposure to TCM, aripiprazole and risperidone, as well as patients who have a higher level of education, were protective factors against the occurrence of MetS. Its clinical value lies in providing a new strategy for the long-term management of MetS that “does not interfere with the control of psychiatric symptoms,” supporting individualized interventions that consider age, different educational backgrounds, and the metabolic risks of antipsychotic drugs. Future research should focus on: ① Conducting multicenter large-sample prospective RCTs to verify the long-term efficacy and safety of TCM combined treatment; ② Using multi-omics technologies (such as metabolomics and gut microbiome sequencing) to analyze the multi-target mechanisms of TCM “AMPK-microbiome-inflammation” and the syndrome-effect relationship; ③ Optimizing TCM prescriptions and clinical application plans based on real-world data, ultimately forming a comprehensive management model of “TCM syndrome differentiation + precise selection of Western medicine + lifestyle intervention” to improve patients’ long-term physical and mental health and reduce cardiovascular risks.

## Data Availability

Our dataset, derived from electronic health records (EHRs) of Xiamen Xianyue Hospital (January 2022–December 2024), is subject to the following restrictions. Access Control: Raw data are stored on password-protected hospital servers and require formal ethics committee approval (XYECC-2024-038) for access, ensuring patient confidentiality. Privacy Protection: Data are de-identified (removal of names, IDs, and exact dates) per GDPR and local regulations, though residual re-identification risks limit public sharing. Usage Scope: Per hospital policy, data may only be used for academic research on psychiatric comorbidities; commercial or non-research applications are prohibited. Temporal/Geographic Limits: Findings are constrained to a single-center, urban Chinese population (Fujian Province), limiting generalizability to other regions or healthcare systems. Requests to access the datasets should be directed to the corresponding author.

## References

[B1] Abel K. M. Drake R. Goldstein J. M. (2010). Sex differences in schizophrenia. Int. Rev. Psychiatry Abingdon, Engl. 22 (5), 417–428. 10.3109/09540261.2010.515205 21047156

[B2] Barton B. B. Zagler A. Engl K. Rihs L. Musil R. (2020). Prevalence of obesity, metabolic syndrome, diabetes and risk of cardiovascular disease in a psychiatric inpatient sample: results of the metabolism in psychiatry (MiP) study. Eur. Archives Psychiatry Clin. Neurosci. 270 (5), 597–609. 10.1007/s00406-019-01043-8 31302731

[B3] Burghardt K. J. Ellingrod V. L. (2013). Detection of metabolic syndrome in schizophrenia and implications for antipsychotic therapy: is there a role for folate? Mol. Diagnosis and Ther. 17 (1), 21–30. 10.1007/s40291-013-0017-8 23341251 PMC4077272

[B4] Carli M. Kolachalam S. Longoni B. Pintaudi A. Baldini M. Aringhieri S. (2021). Atypical antipsychotics and metabolic syndrome: from molecular mechanisms to clinical differences. Pharm. Basel, Switz. 14 (3), 238. 10.3390/ph14030238 33800403 PMC8001502

[B5] Che Y. Yao K. Xi Y. Chen Z. Li Y. Yu N. (2016). Wendan decoction (温胆汤) for treatment of schizophrenia: a systematic review of randomized controlled trials. Chin. J. Integr. Med. 22 (4), 302–310. 10.1007/s11655-015-2047-z 25847776

[B6] Chen M. Yang F. Yang X. Lai X. Gao Y. (2016). Systematic understanding of mechanisms of a Chinese herbal formula in treatment of metabolic syndrome by an integrated pharmacology approach. Int. J. Mol. Sci. 17 (12), 2114. 10.3390/ijms17122114 27999264 PMC5187914

[B7] Chen M. Yang F. Kang J. Gan H. Lai X. Gao Y. (2018). Discovery of molecular mechanism of a clinical herbal formula upregulating serum HDL-c levels in treatment of metabolic syndrome by *in vivo* and computational studies. Bioorg. and Med. Chem. Lett. 28 (2), 174–180. 10.1016/j.bmcl.2017.11.033 29196136

[B8] Chen S. Zeng J. Li R. Zhang Y. Tao Y. Hou Y. (2024). Traditional Chinese medicine in regulating macrophage polarization in immune response of inflammatory diseases. J. Ethnopharmacol. 325, 117838. 10.1016/j.jep.2024.117838 38310986

[B9] Chinese Diabetes Society (2018). The guidelines for the prevention and treatment of type 2 diabetes mellitus in China (2018 edition). Chin. J. Pract. Intern. Med. 38 (4), 292–344.

[B10] Correll C. U. Druss B. G. Lombardo I. O’Gorman C. Harnett J. P. Sanders K. N. (2010). Findings of a U.S. national cardiometabolic screening program among 10,084 psychiatric outpatients. Psychiatr. Serv. Wash. D.C. 61 (9), 892–898. 10.1176/ps.2010.61.9.892 20810587

[B11] Daneshpour M. S. Zarkesh M. Masjoudi S. Azizi F. Hedayati M. (2021). Chromosomal regions strongly associated with waist circumference and body mass index in metabolic syndrome in a family-based study. Sci. Rep. 11 (1), 6082. 10.1038/s41598-021-85741-1 33727680 PMC7966400

[B12] Dang Y. Sun X. Jiang J. (2025). Role of traditional Chinese medicine in pathogen-induced metabolic reprogramming and immune suppression. Sichuan Da Xue Xue Bao. Yi Xue Ban = J. Sichuan Univ. Med. Sci. Ed. 56 (1), 10–18. 40109470 10.12182/20250160501 PMC11914019

[B13] de Boer N. Guloksuz S. van Baal C. Willebrands L. Deenik J. Vinkers C. H. (2021). Study protocol of a randomized, double-blind, placebo-controlled, multi-center trial to treat antipsychotic-induced weight gain: the metformin-lifestyle in antipsychotic users (MELIA) trial. BMC Psychiatry 21 (1), 4. 10.1186/s12888-020-02992-4 33402159 PMC7783702

[B14] De Hert M. Hanssens L. van Winkel R. Wampers M. Van Eyck D. Scheen A. (2007). A case series: evaluation of the metabolic safety of aripiprazole. Schizophr. Bull. 33 (3), 823–830. 10.1093/schbul/sbl037 16940338 PMC2526132

[B15] Ellingrod V. L. Taylor S. F. Dalack G. Grove T. B. Bly M. J. Brook R. D. (2012). Risk factors associated with metabolic syndrome in bipolar and schizophrenia subjects treated with antipsychotics: the role of folate pharmacogenetics. J. Clin. Psychopharmacol. 32 (2), 261–265. 10.1097/JCP.0b013e3182485888 22370993 PMC3622480

[B16] Guo W. Yao X. Cui R. Yang W. Wang L. (2022). Mechanisms of Paeoniaceae action as an antidepressant. Front. Pharmacol. 13, 934199. 10.3389/fphar.2022.934199 36844911 PMC9944447

[B17] Hoveling L. A. Lepe A. Boissonneault M. de Beer J. A. A. Smidt N. de Kroon M. L. A. (2023). Educational inequalities in metabolic syndrome prevalence, timing, and duration amongst adults over the life course: a microsimulation analysis based on the lifelines cohort study. Int. J. Behav. Nutr. Phys. Activity 20 (1), 104. 10.1186/s12966-023-01495-1 37667275 PMC10478481

[B18] Hu X. Zhang Y. Xue Y. Zhang Z. Wang J. (2018). Berberine is a potential therapeutic agent for metabolic syndrome *via* brown adipose tissue activation and metabolism regulation. Am. J. Transl. Res. 10 (11), 3322–3329. 30662589 PMC6291723

[B19] Huang Y.-M. Xu J.-H. Ling W. Li Y. Zhang X.-X. Dai Z.-K. (2015). Efficacy of the wen dan decoction, a Chinese herbal formula, for metabolic syndrome. Altern. Ther. Health Med. 21 (4), 54–67. 26030117

[B20] Jauhar S. Johnstone M. McKenna P. J. (2022). Schizophrenia. Lancet London, Engl. 399 (10323), 473–486. 35093231 10.1016/S0140-6736(21)01730-X

[B21] Jiang X. Jianhua Z. He G. Yan W. Fu K. (2019). The efficacy of ning shen wen dan decoction in treating schizophrenia with predominant negative symptoms and its influence on the content of GABA and glu in plasma. Chin. J. TCM and Pharmacol. 37 (10), 2500–2503.

[B22] Jones M. Jones A. (2008). The effect of antipsychotic medication on metabolic syndrome. Nurs. Stand. R. Coll. Nurs. (Great Britain) 1987 22 (48), 43–48. 10.7748/ns2008.08.22.48.43.c6636 18727355

[B23] Joshi K. B. Nillawar A. Thorat A. P. (2013). Cardiovascular disease risk in schizophrenia patients: a case control study. J. Clin. Diagnostic Res. JCDR 7 (12), 2694–2696. 10.7860/JCDR/2013/7592.3734 24551615 PMC3919370

[B24] Kahl K. G. Greggersen W. Schweiger U. Cordes J. Correll C. U. Frieling H. (2013). Prevalence of the metabolic syndrome in patients with borderline personality disorder: results from a cross-sectional study. Eur. Archives Psychiatry Clin. Neurosci. 263 (3), 205–213. 10.1007/s00406-012-0339-2 22777277

[B25] Kim I. Song Y.-M. Ko H. Sung J. Lee K. Shin J. (2018). Educational disparities in risk for metabolic syndrome. Metabolic Syndrome Relat. Disord. 16 (8), 416–424. 10.1089/met.2017.0170 29975597

[B26] Krijnen H. K. Hoveling L. A. Liefbroer A. C. Bültmann U. Smidt N. (2022). Socioeconomic differences in metabolic syndrome development among males and females, and the mediating role of health literacy and self-management skills. Prev. Med. 161, 107140. 10.1016/j.ypmed.2022.107140 35803357

[B27] Lam L. K. Poon L. Y. Xu P.-L. Xie P.-C. Xie T. Xiao Y. (2024). Efficacy and safety of a Chinese medicine formula diankuang mengxing decoction combined with antipsychotics in the treatment of schizophrenia: a meta-analysis of randomized controlled trials. Medicine 103 (36), e39489. 10.1097/md.0000000000039489 39252273 PMC11384066

[B28] Lee N. Y. Kim S. H. Jung D. C. Kim E. Y. Yu H. Y. Sung K. H. (2011). The prevalence of metabolic syndrome in Korean patients with schizophrenia receiving a monotherapy with aripiprazole, olanzapine or risperidone. Prog. Neuro-Psychopharmacology and Biol. Psychiatry 35 (5), 1273–1278. 10.1016/j.pnpbp.2011.03.022 21513765

[B29] Lee J. S. Kwon J. S. Kim D. Kim S.-W. Kim J.-J. Kim J.-H. (2017). Prevalence of metabolic syndrome in patients with schizophrenia in Korea: a multicenter nationwide cross-sectional study. Psychiatry Investig. 14 (1), 44–50. 10.4306/pi.2017.14.1.44 28096874 PMC5240463

[B30] Lei S. Peng W. Wu L. Yu L. Wang M. Li Q. (2024). Chaihu shugan powder restores fatty acid synthesis to alleviate insulin resistance in metabolic syndrome by regulating the LXRα/SREBP-1 signaling pathway. Front. Pharmacol. 15, 1442279. 10.3389/fphar.2024.1442279 39564113 PMC11573559

[B31] Liu D.-Y. Li Y.-H. Xu Y.-T. Zhu Y. (2016). Anti-aging traditional Chinese medicine: potential mechanisms involving AMPK pathway and calorie restriction based on ‘medicine-food homology’ theory. Zhongguo Zhong Yao Za Zhi = Zhongguo Zhongyao Zazhi = China J. Chin. Materia Medica 41 (6), 1144–1151. 10.4268/cjcmm20160629 28875685

[B32] Liu Z.-C. Fu H.-J. Li N.-C. Deng F.-J. Gan Y.-K. Ye Y.-J. (2025). Systematic pharmacology and experimental validation to elucidate the inflammation-associated mechanism of huanglian wendan (HLWD) decoction in the treatment of MAFLD associated with atherosclerosis. J. Ethnopharmacol. 337 (Pt 1), 118841. 10.1016/j.jep.2024.118841 39299361

[B33] Lu L. Yu X. (2020). Norms for diagnosis and treatment of mental disorders. 2020 edition.

[B34] Ma J. Zhang L. Huang Z. Wang G. (2024). Clinical patterns of metabolic syndrome in young, clinically stable, olanzapine-exposed patients with schizophrenia. Ann. General Psychiatry 23 (1), 46. 10.1186/s12991-024-00532-y 39538330 PMC11562516

[B35] Meng Q. Qi X. Fu Y. Chen Q. Cheng P. Yu X. (2020). Flavonoids extracted from mulberry (Morus Alba L.) leaf improve skeletal muscle mitochondrial function by activating AMPK in type 2 diabetes. J. Ethnopharmacol. 248, 112326. 10.1016/j.jep.2019.112326 31639486

[B36] Moilanen J. Huhtaniska S. Haapea M. Jääskeläinen E. Veijola J. Isohanni M. (2015). Brain morphometry of individuals with schizophrenia with and without antipsychotic medication – the northern Finland birth Cohort 1966 study. Eur. Psychiatry 30 (5), 598–605. 10.1016/j.eurpsy.2015.02.009 25791180

[B37] Mora-Rodriguez R. Ortega J. F. Morales-Palomo F. Ramirez-Jimenez M. (2018). Weight loss but not gains in cardiorespiratory fitness after exercise-training predicts improved health risk factors in metabolic syndrome. Nutr. Metabolism, Cardiovasc. Dis. NMCD 28 (12), 1267–1274. 10.1016/j.numecd.2018.08.004 30459053

[B38] Pan Y. Kong L.-D. (2018). High fructose diet-induced metabolic syndrome: pathophysiological mechanism and treatment by traditional Chinese medicine. Pharmacol. Res. 130, 438–450. 10.1016/j.phrs.2018.02.020 29471102

[B39] Pan X. Yin X. Li K. (2021). The efficacy of modified ‘Dian Kuang Meng Xing Tang’ combined with aripiprazole in the treatment of schizophrenia with liver depression and phlegm obstruction, and its effects on lipid metabolism and high-sensitivity C-reactive protein. Chin. J. Traditional Chin. Med. 39 (12), 175–178.

[B40] Paredes R. M. Quinones M. Marballi K. Gao X. Valdez C. Ahuja S. S. (2014). Metabolomic profiling of schizophrenia patients at risk for metabolic syndrome. Int. J. Neuropsychopharmacol. 17 (8), 1139–1148. 10.1017/S1461145714000157 24565079

[B41] Petrova N. N. Semenova N. V. (2024). Metabolic syndrome and antipsychotic therapy of schizophrenia. Zhurnal Nevrol. I Psikhiatrii Im. S.S. Korsakova 124 (11), 165–170. 10.17116/jnevro2024124111165 39690565

[B42] Rathbone J. Zhang L. Zhang M. Xia J. Liu X. Yang Y. (2007). Chinese herbal medicine for schizophrenia: cochrane systematic review of randomised trials. Br. J. Psychiatry J. Ment. Sci. 190, 379–384. 10.1192/bjp.bp.106.026880 17470951

[B43] Reyes-Ortiz C. A. Marín-Vargas E. Ocampo-Chaparro J. M. (2024). Social determinants of health and metabolic syndrome in Colombian older adults. Nutr. Metabolism, Cardiovasc. Dis. NMCD 34 (7), 1751–1760. 38413358 10.1016/j.numecd.2024.01.022

[B44] Rojo L. E. Gaspar P. A. Silva H. Risco L. Arena P. Cubillos-Robles K. (2015). Metabolic syndrome and obesity among users of second generation antipsychotics: a global challenge for modern psychopharmacology. Pharmacol. Res. 101, 74–85. 10.1016/j.phrs.2015.07.022 26218604

[B45] Saari K. M. Lindeman S. M. Viilo K. M. Isohanni M. K. Järvelin M.-R. Laurén L. H. (2005). A 4-fold risk of metabolic syndrome in patients with schizophrenia: the northern Finland 1966 birth cohort study. J. Clin. Psychiatry 66 (5), 559–563. 10.4088/jcp.v66n0503 15889940

[B46] Saddichha S. Manjunatha N. Ameen S. Akhtar S. (2008). Metabolic syndrome in first episode schizophrenia—A randomized double-blind controlled, short-term prospective study. Schizophrenia Res. 101 (1–3), 266–272. 10.1016/j.schres.2008.01.004 18262771

[B47] Saloojee S. Burns J. K. Motala A. A. (2016). Metabolic syndrome in South African patients with severe mental illness: prevalence and associated risk factors. PloS One 11 (2), e0149209. 10.1371/journal.pone.0149209 26882230 PMC4755575

[B48] Scaini G. Quevedo J. Velligan D. Roberts D. L. Raventos H. Walss-Bass C. (2018). Second generation antipsychotic-induced mitochondrial alterations: implications for increased risk of metabolic syndrome in patients with schizophrenia. Eur. Neuropsychopharmacol. J. Eur. Coll. Neuropsychopharmacol. 28 (3), 369–380. 10.1016/j.euroneuro.2018.01.004 29449054

[B49] Schreiner A. Niehaus D. Shuriquie N. A. Aadamsoo K. Korcsog P. Salinas R. (2012). Metabolic effects of paliperidone extended release *versus* oral olanzapine in patients with schizophrenia: a prospective, randomized, controlled trial. J. Clin. Psychopharmacol. 32 (4), 449–457. 10.1097/JCP.0b013e31825cccad 22722501

[B50] Shi N. Zhou Y. Ma H. (2022). A network pharmacology study of mechanism and efficacy of jiawei huanglian-wendan decoction in polycystic ovary syndrome with insulin resistance. Medicine 101 (48), e32057. 10.1097/MD.0000000000032057 36482532 PMC9726404

[B51] Shojaeimotlagh V. Hashiehbaf A. Karami M. Monjazebi F. Gheshlagh R. G. (2019). Prevalence of metabolic syndrome in Iranian patients with schizophrenia: a systematic review and meta-analysis. Diabetes and Metabolic Syndrome 13 (1), 143–147. 10.1016/j.dsx.2018.08.014 30641687

[B52] Sommer I. E. Tiihonen J. van Mourik A. Tanskanen A. Taipale H. (2020). The clinical course of schizophrenia in women and men-a nation-wide cohort study. NPJ Schizophr. 6 (1), 12. 10.1038/s41537-020-0102-z 32358572 PMC7195359

[B53] Subashini R. Deepa M. Padmavati R. Thara R. Mohan V. (2011). Prevalence of diabetes, obesity, and metabolic syndrome in subjects with and without schizophrenia (CURES-104). J. Postgrad. Med. 57 (4), 272–277. 10.4103/0022-3859.90075 22120854

[B54] Tao H. Shen D. Zhou Y. Sun F. Li G. Jin W. (2022). A systematic review and meta-analysis of metabolic syndrome prevalence in Chinese inpatients with bipolar disorder. Hormone Metabolic Res. = Hormon- Und Stoffwechselforschung = Hormones Metabolisme 54 (9), 587–592. 10.1055/a-1882-8423 35738391

[B55] Tian E. Sharma G. Dai C. (2023). Neuroprotective properties of berberine: molecular mechanisms and clinical implications. Antioxidants Basel, Switz. 12 (10), 1883. 10.3390/antiox12101883 37891961 PMC10604532

[B56] Tian Y. Pang G. Pan L. (2023). Clinical efficacy of huanglian Wendan decoction in treating type 2 diabetes mellitus: a systematic review and meta-analysis. Medicine 102 (40), e35299. 10.1097/MD.0000000000035299 37800822 PMC10553187

[B57] Tocco M. Newcomer J. W. Mao Y. Pikalov A. Loebel A. (2021). Lurasidone and risk for metabolic syndrome: results from short- and long-term clinical studies in patients with schizophrenia. CNS Spectrums 26 (6), 614–624. 10.1017/S1092852920001698 32921337

[B58] Tschoner A. Engl J. Rettenbacher M. Edlinger M. Kaser S. Tatarczyk T. (2009). Effects of six second generation antipsychotics on body weight and metabolism—Risk assessment and results from a prospective study. Pharmacopsychiatry 42 (1), 29–34. 10.1055/s-0028-1100425 19153944

[B59] Vancampfort D. Stubbs B. Mitchell A. J. De Hert M. Wampers M. Ward P. B. (2015). Risk of metabolic syndrome and its components in people with schizophrenia and related psychotic disorders, bipolar disorder and major depressive disorder: a systematic review and meta-analysis. World Psychiatry Official J. World Psychiatric Assoc. (WPA) 14 (3), 339–347. 10.1002/wps.20252 26407790 PMC4592657

[B60] Ventriglio A. Baldessarini R. J. Vitrani G. Bonfitto I. Cecere A. C. Rinaldi A. (2018). Metabolic syndrome in psychotic disorder patients treated with oral and long-acting injected antipsychotics. Front. Psychiatry 9, 744. 10.3389/fpsyt.2018.00744 30700975 PMC6343459

[B61] Wampers M. Hanssens L. van Winkel R. Heald A. Collette J. Peuskens J. (2012). Differential effects of olanzapine and risperidone on plasma adiponectin levels over time: results from a 3-month prospective open-label study. Eur. Neuropsychopharmacol. J. Eur. Coll. Neuropsychopharmacol. 22 (1), 17–26. 10.1016/j.euroneuro.2011.03.010 21511441

[B62] Wu Y. C. Hill R. A. Gogos A. van den Buuse M. (2013). Sex differences and the role of estrogen in animal models of schizophrenia: interaction with BDNF. Neuroscience 239, 67–83. 10.1016/j.neuroscience.2012.10.024 23085218

[B63] Wu H. Tian J. Dai D. Liao J. Wang X. Wei X. (2020). Efficacy and safety assessment of traditional Chinese medicine for metabolic syndrome. BMJ Open Diabetes Res. and Care 8 (1), e001181. 10.1136/bmjdrc-2020-001181 32220922 PMC7170408

[B64] Wu Q. Liu J. Mao Z. Tian L. Wang N. Wang G. (2022). Ligustilide attenuates ischemic stroke injury by promoting Drp1-mediated mitochondrial fission *via* activation of AMPK. Phytomedicine Int. J. Phytotherapy Phytopharm. 95, 153884. 10.1016/j.phymed.2021.153884 34929562

[B65] Xu N. Ijaz M. Shu Y. Wang P. Ma L. Wang P. (2024). The *in vivo* study on antioxidant activity of wendan decoction in treating hyperlipidemia: a pharmacokinetic-pharmacodynamic (PK-PD) model. Front. Pharmacol. 15, 1260603. 10.3389/fphar.2024.1260603 38323083 PMC10844532

[B66] Yumru M. Savas H. A. Kurt E. Kaya M. C. Selek S. Savas E. (2007). Atypical antipsychotics related metabolic syndrome in bipolar patients. J. Affect. Disord. 98 (3), 247–252. 10.1016/j.jad.2006.08.009 16970993

[B67] Zhang Y. Dai G. (2012). Efficacy and metabolic influence of paliperidone ER, aripiprazole and ziprasidone to patients with first-episode schizophrenia through 52 weeks follow-up in China. Hum. Psychopharmacol. 27 (6), 605–614. 10.1002/hup.2270 24446539

[B68] Zhang L. Li J. Zhao Y. Su Y. Si T. (2016). Critical evaluation of paliperidone in the treatment of schizophrenia in Chinese patients: a systematic literature review. Neuropsychiatric Dis. Treat. 12, 113–131. 10.2147/NDT.S64672 26811684 PMC4714741

[B69] Zhang Y. Wang Q. Reynolds G. P. Yue W. Deng W. (2020). Metabolic effects of 7 antipsychotics on patients with schizophrenia: a Short-Term, randomized, Open-label, multicenter, pharmacologic trial. J. Clin. Psychiatry 81 (3), 19m12785. 10.4088/JCP.19m12785 32237292

[B70] Zhao S. Hao R. Zhao J. Ma K. Li J. Tian C. (2024). Efficacy and safety of combined Chinese and Western medicine in the treatment of metabolic syndrome: a network meta-analysis of randomized controlled trials. Heliyon 10 (16), e35811. 10.1016/j.heliyon.2024.e35811 39224309 PMC11366876

[B71] Zhou S. Xu H. Zhu J. Fan X. Zhang J. (2023). Clinical efficacy and metabolomics study of wendan decoction in the treatment of phlegm-dampness obstructive sleep apnea-hypopnea syndrome with type 2 diabetes mellitus. J. Ethnopharmacol. 317, 116775. 10.1016/j.jep.2023.116775 37311503

